# Hypertension Cascade Across Three Healthcare Systems and in Relation to the Level of Implementation of the Integrated Care Package

**DOI:** 10.5334/ijic.8921

**Published:** 2025-08-22

**Authors:** Veerle Buffel, Philippe Bos, Savina Chham, Srean Chimm, Katrien Danhieux, Grace Marie Ku, Josefien Van Olmen, Crt Zavrnik, Zalika Klemenc-Ketis, Edwin Wouters

**Affiliations:** 1 Department of Sociology, Brussels Institute of Social and Population Studies (BRISPO), Interface Demography (ID), Vrije Universiteit Brussel (VUB), Brussels, Belgium; 2 Department of Sociology, Centre for Population, Family and Health, University of Antwerp, Antwerp, Belgium; 3 Center for Health System Research and Policy Support, National Institute of Public health, Phnom Penh, Cambodia; 4 School of Public Health, National Institute of Public health, Phnom Penh, Cambodia; 5 Department of Family Medicine and Population Health, University of Antwerp, Wilrijk, Belgium; 6 Division Department of Public Health, Organization Institute of Tropical Medicine, Antwerp, Belgium; 7 Faculty of Medicine & Pharmacy, Vrije Universiteit Brussel, Brussels, Belgium; 8 Faculty of Medicine & Surgery, University of Santo Tomas, Manila, Philippines; 9 Community Health Centre Ljubljana, Primary Health care Research and Development Institute, Ljubljana, Slovenia

**Keywords:** hypertension cascade of care, integrated care package, health care and primary health care systems, socioeconomic vulnerable patients

## Abstract

**Introduction:**

We built Cascades of Care (CoC) for hypertension in Belgium, Slovenia and Cambodia, and assessed CoC stratifications across patients’ gender and socioeconomic status. Differences between the CoCs were studied by looking at the level of implementation of the integrated care package and other health system characteristics.

**Methods:**

A mixed methods design: Age-standardized gender-specific hypertension cascades were built from survey and register data and logistic regression analyses were performed. Focus group discussions with experts were used to interpret these results.

**Results:**

In Belgium, the largest gap is between ‘prevalence’ and ‘diagnosis’. In Cambodia, a large drop –especially among men– is found at the beginning and the end of the cascade. In Slovenia, only a limited number of patients is tested and linked to care, but once registered, attrition is quite low. Poor financial situation was a significant determinant of drop-out across the countries but at different stages of the CoC, and especially in Cambodia large gender differences were observed with women being better retained throughout the CoC.

**Discussion and conclusion:**

Despite contextual differences between the countries and difficulties in comparability of the cascades, lessons can be learnt from each country’s strengths and weaknesses to improve quality of integrated hypertension care.

## Background

Hypertension (HTN) has a global prevalence of 31.1% reflecting its dominance in the global burden of disease [1,2]. Despite a large body of evidence demonstrating that effective treatment exists and is cost-effective [3,4], significant gaps in HTN awareness, treatment and control remain [5]. Moreover, the losses throughout the continuum of HTN care are often gender specific [6,7] and higher among socioeconomically vulnerable patients [8,9]. However, the manifestations of these gender differences and socioeconomic inequalities in HTN management are inconsistent and vary across countries [5,6,7,8,9].

HTN is diagnosed and managed largely at the primary care (PC) level. Entailing a combination of sustained lifestyle changes and medication, successful treatment requires continuous monitoring and interactions with the health care (HC) system [10]. There is strong evidence that integrated care leads to improved care processes, to HC that is responsive to people’s needs and to better health outcomes [11,12,13]. Integrated care can be described as a coherent and coordinated set of services planned, managed and offered to individual service users by a number of organisations and a range of cooperating professionals and informal carers [14]. The World Health Organization (WHO) [15] integrated care package (ICP) for HTN recommendations and guidelines consists of six key elements for the management of HTN care [16]: (a) early detection and diagnosis of people with HTN and subsequent (b) treatment in PC services, (c) health education and (d) self-management support to patients and caregivers, (e) collaboration between caregivers, and (f) coordinated organization of care. However, the level of ICP implementation varies across HC systems [14,17] and how this is related to differences in the quality of the HTN care continuum remains unclear.

The objective of this study was three-fold: (1) to estimate and compare gender specific HTN Cascades of Care (CoCs) in three different HC systems; (2) to assess the impact of patients’ socioeconomic status on reaching the various steps of the CoC; and (3) to interpret the differences between the CoCs in relation to the implementation of the integrated care elements and other HC characteristics.

Recently, the number of studies using a CoC approach for HTN has grown. However, most of these studies [5,8,9,18,19,20] rely on a basic CoC that includes only the steps ‘awareness’, ‘treatment’ and ‘control’ [10]. Calls have been made to adopt a more expanded cascade that incorporates structural- and process-quality indicators, enabling a more accurate assessment of HC system performance [10].

In this study, we employed an expanded CoC approach, which also includes the steps ‘screening’, ‘in care’ and ‘treatment adherence’. Additionally, we linked the steps to structural quality indicators, operationalized as the level of implementation of the ICP elements. We compared HTN CoCs across three countries: two high-income European countries (Belgium and Slovenia) and one lower-middle income Asian country (Cambodia). This approach moves beyond the many single-country studies [8,18,19,20], offering deeper insights into the impact of different HC system characteristics on the HTN CoC and potential stratification by gender and socioeconomic status. Although our comparison is limited to three countries –unlike existing multi-country comparisons [5,9,21]– this narrower scope enables an in-depth analysis. Furthermore, we consulted country-specific experts to contextualize observed differences in light of ICP implementation levels.

We selected these three countries to capture different HC and first line health systems (FLHS) [22]. To systematically and comparably describe these systems, we relied on the work of Reibling [23] and Kringos [24,25]. Reibling [23] developed a HC system typology, incorporating indicators that assess the role of PC within the broader HC system (see supplementary material S1.a). Although the typology was initially developed for OECD countries, the indicators can also be used in the Cambodian context. The work of Kringos [24,25] fits only for Belgium and Slovenia. He measured the strength of dimensions of the PC system (see S1.b). Additional information on the socioeconomic and demographic profiles of the three countries is provided in supplementary file S1.c.

Belgium and Slovenia have a supply- and choice-oriented public system, characterized by a medium to high level of financial and human resources [23]. In Cambodia, the level of financial and human resources is much lower and comes especially from domestic private sources. In relative terms, the expenditure on the FLHS is low in Belgium and Slovenia, while very high in Cambodia, which has also a significant high ratio of General practitioners (GP) to specialists (higher than the EU maximum).

While both Belgium and Slovenia have PC as their FLHS; they display important differences. Belgium has a privatized HC system, which is funded through a mix of direct government payment and refunding of patients through third party payers. HC providers and patients enjoy a high degree of autonomy in their choice of service utilisation and choice of HC provider, which has led to a fragmented system of individualized care. According to Kringos’ [24,25] operationalization; the Belgian HC system is only moderately PC oriented, with a weak score on accessibility and median scores on the governance of the PC system, workforce development and coordination dimension.

The Slovenian HC system is based on the Bismarck model with a diversified revenue base and the introduction of (some) private providers of HC services. About 76% of PC level physicians practice in community health centres throughout the country, while others practice privately (so-called concessionaires), but under contract with the Health Insurance Institute of Slovenia. The overall PC system is considered to be strong in the work of Kringos [24,25] but with weak scores on continuity and comprehensiveness.

Finally, Cambodia has a mixed HC system of both public and private health providers, and has PC as its FLHS. The public sector is the prominent provider of preventive services and in-patient admission, whereas the private sector tends to dominate provision of outpatient curative consultation [26]. To date, the public HC intervention for HTN has been concentrated at the primary health systems level, so-called ‘operational districts’, and delivered in three platforms: the NCD clinic at the Referral Hospitals, the WHO package of essential non-communicable diseases intervention (PEN) at the Health Centre and community-based care under the MoPoTsyo network [27]. Despite the three public health platforms for NCD intervention, there are a number of private health providers offering HTN care.

## Methods

### Study setting and design

This observational study was conducted as part of a larger international project ‘Scaling up an integrated diabetes and HTN care package for vulnerable people at risk in Belgium, Slovenia and Cambodia’ (SCUBY) [22]. The first phase of the project focused on constructing country-specific CoCs, quantifying losses at each stage, and relating these losses to patient characteristics [27,28]. The second phase involved both country-specific evaluations [28,29,30] and a cross-national assessment of ICP implementation [17]. The present study builds on this foundation to better understand differences in gender-specific CoCs and their stratification by socioeconomic status, considering HC system characteristics and the level of ICP implementation.

We employed a mixed-methods approach using a sequential explanatory design. This approach began with quantitative analyses, followed by qualitative data collection and analysis, to enable a more comprehensive interpretation of the results [32]. First, we developed gender specific CoCs across the three countries, as comparable as possible, in light of existing data and guidelines for HTN care. Second, we performed multivariate logistic regression analyses to examine whether gender and socioeconomic status were associated with losses at different stages of the HTN CoC, controlling for other individual characteristics. Third, within multidisciplinary focus group discussions (FGD), we compared and interpreted the results of the HTN CoCs by looking at the HC system characteristics and level of ICP implementation.

### Quantitative data and measures

#### The cascades

As the three countries have different health information systems, we used different types of data to estimate the CoCs: representative survey data for Belgium and Cambodia, and administrative data for Slovenia. In line with prior research [33] and following consensus on the target age-group of guidelines for the management of HTN in the three countries [34,35,36], the samples were restricted to individuals aged 40–79 years old.

For Belgium we relied on data of the 2018 Belgian Health Interview Survey (BHIS) [37] and the Belgium Health Examination Survey (BHES) [38]. The BHIS is a recurring cross-sectional survey collecting self-reported health information among a representative sample of the Belgian population, while the BHES collected additional health information through clinical examinations and analyses of biological samples among a subsample of BHIS respondents (N people aged 40–79 = 828) [38].

In Slovenia, treatment of HTN at the PC level is supported through a clinical protocol, integrated with the health information system. Individual data from HTN-specific check-ups were thus available; the 2019 data were used for the analysis. However, the data are not managed at the national level; we therefore used data from the Community Health Centre Ljubljana (CHCL). CHCL is the largest primary health centre in Slovenia and includes 120 practices with more than 300 000 patients covering both urban, suburban and, to some extent, the rural areas, covering about 20% of the Slovenian population.

In Cambodia, we relied on population-based data collected through a household survey in 2019–2020 among people aged ≤40 years. The survey was conducted in five purposively selected Operational Districts (OD) with different combinations of health system interventions (hospital-based; health centre-based; community based and private care). Within each OD, 44 villages were randomly selected using the probability-proportional-to-size sampling method. The selected subsample for the current study consists of 5070 respondents between 40 and 79 years old [39].

The measures used for the three country CoCs are presented in Table 1. These are in consonance with other HTN cascade studies [10].

**Table 1:** Operationalization of the bars of the Cascade per country.


BAR	BELGIUM	SLOVENIA	CAMBODIA	NOTES

**Prevalence**	based on survey measuring SBP ≥140 mmHg OR a DBP ≥ 90 mmH^a^ OR self-reported use of antihypertensive medication during the past two weeks OR self-reported diagnosis	no correct estimation possible of the number of people with ‘undiagnosed HTN’ and thus not of the ‘prevalence’	based on a survey measuring SBP ≥ 140 mmHg OR DBP ≥ 90 mmHg OR self-reported diagnosis as having been told by healthcare professionals that they have HTN	Denominator: total population aged 40–79

**Tested**	reported that they had a BP measurement in the last 3 years	BP measure by registered nurse in the last 3 years	reported that they had a BP measurement in the last 3 years	

**1. Diagnosed**	reported that they have the condition ‘HTN’	registered as ‘having the diagnosis HTN’	reported that they were diagnosed with HTN by a healthcare professional	the cascade will start from the ‘diagnosed’ bar, with diagnosed as ‘100%’

**2. In care**	followed by a healthcare professional for HTN during the past 12 months	HTN consult. in the past 12 months	get treatment/care for HTN in the past 12 months	

**3. In treatment**	either self-reported use of medication or following a diet to treat HTN during the past 12 months	at least one BP measure in the last 12 months	currently receiving treatment/advice for HTN prescribed by a doctor or other HC worker: Drugs (2w) /diet advice (reduce salt/lose weight/physical exercise.)	in SL there is no information available about HTN medication or non-medical treatment; because BP measure is part of the treatment protocol of HTN, this indicator is used as a proxy of treatment

**4. Adhered to treatment (medication adherence)**	took prescribed HTN medication last 24h (‘yes’) AND regularly (‘yes’)	adherence assessment HTN: regularly (‘yes’) AND properly (‘yes’)	MARS-5 adherence scale for HTN medication: high adherence (vs. no)^b^	only among patients who took HTN medication

**5. Under control**	having SBP <140 mmHg and DBP <90 mmHg	having SBP <140 mmHg and DBP <90 mmHg	having SBP <140 mmHg and DBP <90 mmHg	


*Notes:* ^a^BP was measured by trained nurses during a home visit as part of the BHES fieldwork. Respondent’s SBP and DBP were determined by taking the respective averages of the last two out of three BP measurement [38]. ^b^ Participants indicate how often they engage in each of five HTN medication-adherent behaviours (e.g. “I take less than instructed”) on a 1–5 likert scale (always to never). The item scores are summed to indicate overall level of adherence. Consistent with previous research [40,41], MARS scores were then dichotomised to give low adherence (LAd, MARS score ≤21) and high adherence sub-groups (HAd, MARS score N21).

Socioeconomic vulnerability was defined using two indicators: low educational level and poor financial situation. Low educational level was operationalized as having completed primary education or less. Poor financial situation was defined differently across the settings: in Belgium and Slovenia, it referred to experiencing financial problems, while in Cambodia, it corresponded to the lowest two categories of the wealth quintile. In addition, we take age, gender, body mass index, smoking status and comorbidity diabetes type 2 (T2D) into account. Detailed information about the operationalization of these determinants can be found in S2.

#### Level of implementation of integrated care

**Figure 1:**
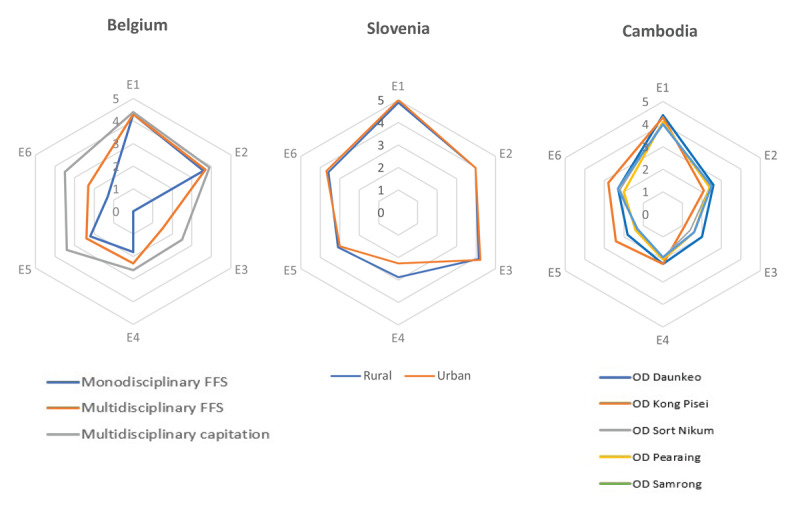
ICP grid scores per country. *Notes:* E1:Identification, E2:Treatment, E3:Health education, E4:Self-management support, E5:Structured collaboration, E6:Organization of care; the medium scores are presented; see S6.a-c for the corresponding data. The elements are based on several items and operationalized as scales ranging from 0 (no implementation) to 5 (complete implementation). FFS = fee-for-service; OD = operational district; corresponding numbers in table S4.a-c.

Data on the level of implementation of integrated care were collected by the SCUBY project team at the HC facility level through structured interviews conducted in 2019–2020. More detailed information about the data collection and sampling frame can be found in S3 and elsewhere [17,29,30,31]. The level of ICP implementation was measured using the ICP Grid, which comprises six elements [16,59]: identification, treatment, health education, self-management, structural collaboration and organization of care. ICP grid scores are statistically described (see tables in S4.a-c) and visualized using radar charts (Figure 1).

### Quantitative analyses

For all three countries, we estimated the age-standardized prevalence of HTN, the proportion that is tested and the proportion whose condition of HTN was diagnosed. For Belgium and Cambodia, we also calculated the age-standardized proportion of individuals with HTN who were tested and diagnosed This was not feasible for Slovenia, as it was not possible to estimate the prevalence of HTN. Among individuals with diagnosed HTN, we estimated the age-standardized proportion of individuals who were ‘in care’, ‘in treatment’, ‘adherent to treatment’ and ‘under control’. We applied a conditional cascade approach and derived a reference population for direct age-standardization of prevalence data by calculating an average proportion in each 10-year age group across all countries included in the analysis, using population estimates for the year 2000 [42].

Logistic regression analyses were performed to estimate the effect of gender and socioeconomic vulnerability on ‘in care’, ‘in treatment’, ‘adherent to treatment’ and ‘under control’.

### Qualitative data collection and analysis

Using purposive sampling, we organized three online FGDs with experts specializing in the organization of chronic care and PC in each country (S5). Experts were recruited through the professional network of the project team. To guide these discussions, a PowerPoint presentation was used, summarizing the constructed HTN CoCs and the results of multivariate regression analyses on the impact of gender and socioeconomic vulnerability on dropouts. Additionally, country-specific ICP Grid scores (Figure 1 and S4a-c) and data on selected HC system indicators (see study setting, S1.a-b) were presented to help interpret differences in the HTN CoCs.

The moderator facilitated the discussions by asking the experts to relate variations in HC systems and the degree of integration in care to the HTN CoCs and observed ender and socioeconomic differences. The discussions were recorded, transcribed, and thematically analysed, using the CoC stages as initial themes.

Preliminary findings from the FGDs were shared with the experts to ensure interpretations aligned with their perspectives. The results were then combined with the quantitative findings and shared in a final written consultation round, allowing experts to provide additional feedback or insights.

## Results

### Quantitative results

Table 2 presents the age-standardised prevalence of HTN and the percentages of people tested and diagnosed with HTN. The prevalence of HTN is very high in Belgium: 42% among men and 36% among women. In Cambodia the prevalence among men is almost 10 percentage points lower.

**Table 2:** The prevalence of HTN, the percentage of people tested and diagnosed with HTN among those aged 40–79 years.


AMONG INDIVIDUALS AGED 40–79															

	PREVALENCE (%)					TESTED (%)					DIAGNOSED (%)				
		
MEN			WOMEN			MEN			WOMEN			MEN			WOMEN		
		
	%	% ASR	%	% ASR	SIG.^b^	%	% ASR	%	% ASR	SIG.	%	% ASR	%	% ASR	SIG.

BE	44.24	41.79	40.97	35.94	n.s.	93.03	92.51	94.27	93.66	n.s.	24.4	22.98	24.87	21.43	n.s.

SL	n.a.^a^		n.a.^a^			23.40	23.04	11.49	11.76	<0.001	15.65	13.99	9.97	8.07	

CA	31.41	30.79	35.13	32.51	n.s.	48.73	48.16	67.29	66.35	<0.001	13.48	12.67	26.4	23.91	

**AMONG INDIVIDUALS WITH HTN AGED 40–79**															

BE						95.73	96.58	97.03	94.62	n.s.	42.1	42.45	53.02	49.32	n.s.

SL						n.a.^a^		n.a.^a^			n.a.^a^		n.a.^a^		

CA						68.13	64.54	87.76	85.21	<0.001	42.45	32.5	75.16	69.32	<0.001


*Notes:* BE = Belgium, SL = Slovenia, CA = Cambodia; ASR = age-standardized rate; n.a. = not available; n.s. = not significant; ^a^no valid estimations of HTN prevalence available for Slovenia and thus also not for ‘being tested’ and ‘diagnosed’ among those with HTN; ^b^The p-value is obtained by a logistic regression model.

The number of people in Belgium who had blood pressure measured in the last three years is very high (men: 92.51%; women: 93.66%) and the percentages are almost 100% among people with HTN. Slovenia reports far lower rates, with BP measurements recorded for less than a quarter of men (23.04%) and only 11.76% of women. In Cambodia, the proportion of women with HTN who were tested is relatively high (85.21%) and considerably higher than that of men (64.54%).

Despite high testing rates in Belgium, people diagnosed with HTN among those with the condition is relatively low (men: 69.32%; women: 49.32%). In Cambodia, the proportion of the hypertensive population diagnosed with HTN is even higher for women (69.32%) compared to Belgium (49.32%).

In Slovenia, the percentage of men diagnosed with HTN (13.99%) is comparable to Cambodia (12.67%), while the percentage for women is much lower (8.07% in Slovenia versus 23.91% in Cambodia). However, as valid estimates of HTN prevalence are unavailable for Slovenia, it remains unclear whether these findings are due to a lower prevalence of HTN or low testing rates.

**Figure 2:**
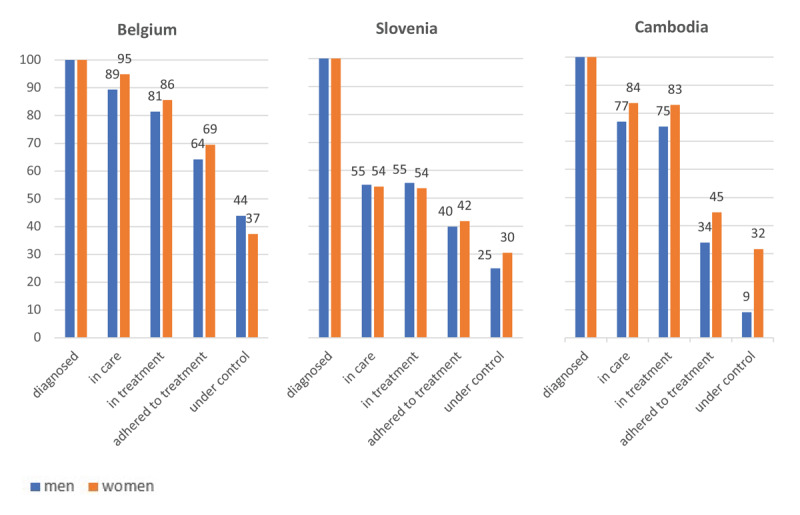
Gender stratified cascade of hypertension. *Note:* The presented scores are age-standardised; see S6 for the corresponding data.

Figure 2 illustrates the gender-stratified cascades per country, starting with patients diagnosed with or aware of HTN. In Belgium, the proportion of male and female HTN patients ‘in care’ (respectively 89.29% and 94.86%) and ‘in treatment’ (81.38% and 85.57%) are very high. In Slovenia, the cascade shows a significant drop, with just over half of the diagnosed population being ‘in care’ (men: 54.84%; women: 54.17%), representing the largest loss in the HTN CoC. In Cambodia, the proportions of patients ‘in care’ (men: 77.03%; women: 84.64%) and ‘in treatment’ (men: 75.26%; women: 82.99%) are also high, similar to Belgium.

In contrast to the Slovenian CoC, the gaps are larger at the end of the cascade in Belgium and Cambodia. In Cambodia, the largest drop is from ‘in treatment’ (men: 75.26%; women: 82.99%) to ‘adherent to treatment’ (40.36%; 44.70%); in Belgium, this is from ‘adherent to treatment’ (68.81%; 69.48%) to ‘controlled’ (43.89%; 37.36%).

Table 3 shows the crude and adjusted odds ratio’s (OR) of the different stages in the cascade for gender, education and financial status, retrieved from the logistic regression analysis. The gender stratification of the HTN cascade was minimal and not significant in Belgium. In contrast, a clear gender stratification was observed in Cambodia. In Slovenia, gender differences were noted in the percentage of individuals tested and diagnosed with HTN (Table 2), with higher proportions among men. However, diagnosed women were slightly more likely to be ‘in care’ and ‘under control’, also after adjusting for other individual characteristics (Table 3). In Cambodia, women were more likely to reach each step of the cascade, starting from ‘tested’ and ‘diagnosed’ (Table 2) to ‘under control’ (Table 3). The only exception was for ‘in treatment’, but this was due to the limited attrition between ‘linked to care’ and ‘in treatment’.

**Table 3:** Gender, education and financial situation regressed on the different bars of the cascade, using a logistic regression analysis (crude and adjusted odds ratios are presented).


BELGIUM																				

	IN CARE = 1 (AMONG THE DIAGNOSED; N = 1137)										IN TREATMENT = 1 (AMONG THE LINKED TO CARE; N = 1080)									
	
	CUDE OR	CI-95		P-VALUE		AOR	CI-95		P-VALUE		CRUDE OR	CI-95		P-VALUE		AOR	CI-95		P-VALUE	

**Gende**r (ref. men)																				

women	1.96	0.94	4.08	0.072		1.84	0.88	3.86	0.105		0.91	0.50	1.64	0.745		1.20	0.67	2.13	0.545	

**Education** (ref. high (higher secondary or higher))																				

low (primary school or lower)	1.37	0.42	4.51	0.606		0.79	0.23	2.65	0.699		0.69	0.30	1.57	0.375		0.60	0.25	1.44	0.255	

middle (lower secondary school)	1.55	0.52	4.63	0.434		1.08	0.35	3.29	0.896		0.91	0.45	1.86	0.796		0.77	0.39	1.53	0.458	

**Financial situation** (ref. good)																				

poor	1.06	0.45	2.5	0.891		1.00	0.41	2.47	0.992		0.42	0.20	0.87	0.02	*	0.37	0.18	0.77	0.008	**

moderate	1.3	0.52	3.27	0.576		1.01	0.39	2.60	0.983		0.47	0.21	1.05	0.067		0.42	0.20	0.88	0.022	*

	**ADHERENT TO TREATMENT = 1 (AMONG THOSE IN TREATMENT; N = 1042)**										**UNDER CONTROL = 1 (AMONG THOSE ADHERED TO TREATMENT)** ^a^									

	**CRUDE OR**	**CI-95**		**P-VALUE**		**ADJUSTED OR**	**CI-95**		**P-VALUE**											

**Gender** (ref. men)																				

women	1.38	0.83	2.28	0.215		1.48	0.86	2.54	0.155											

**Education** (ref. high (higher secondary or higher))																				

low (primary school or lower)	0.62	0.29	1.33	0.221		0.37	0.16	0.83	0.016	*										

middle (lower secondary school)	1.12	0.60	2.08	0.715		0.82	0.42	1.58	0.552											

**Financial situation** (ref. good)																				

poor	1.31	0.75	2.29	0.345		1.25	0.71	2.20	0.445											

moderate	1.21	0.64	2.27	0.554		1.01	0.53	1.90	0.984											

**SLOVENIA**																				

	**IN CARE = 1 (AMONG THE DIAGNOSED; N = 22837)** ^b^										**IN TREATMENT = 1 (AMONG THE LINKED TO CARE; N = 13061)** ^b^									
	
	**CUDE OR**	**CI-95**		**P-VALUE**		**AOR**		**CI-95**	**P-VALUE**		**CRUDE OR**	**CI-95**		**P-VALUE**			**AOR**	**CI-95**	**P-VALUE**	

**Gende**r (ref. men)																				

women	1.09	1.04	1.15	0.001	**	1.08	1.02	1.14	0.005	**	1.36	0.96	1.92	0.082		1.29	0.91	1.83	0.153	

**Education**	//			//							//					//				

**Financial situation**	//			//							//					//				

	**ADHERENT TO TREATMENT = 1 (AMONG THOSE IN TREATMENT; N = 2051)**										**UNDER CONTROL = 1 (AMONG THOSE ADHERED TO CARE; N = 1873)**									
	
	**CRUDE OR**	**CI-95**		**P-VALUE**		**AOR**	**CI-95**	**P-VALUE**			**CRUDE OR**	**CI-95**		**P-VALUE**			**AOR**	**CI-95**	**P-VALUE**	

**Gende**r (ref. men)																				

women	1.26	0.93	1.72	0.143		1.25	0.91	1.74	0.173		1.29	1.07	1.54	0.007	**	1.31	1.08	1.59	0.006	**

**Education** (ref. high (higher secondary or higher))																				

low (primary school or lower)	1.11	0.74	1.74	0.620		1.18	0.76	1.88	0.470		1.12	0.88	1.45	0.359		1.09	0.84	1.42	0.497	

middle (lower secondary school)	1.21	0.84	1.78	0.322		1.26	0.87	1.87	0.231		1.04	0.84	1.29	0.747		1.07	0.86	1.34	0.518	

**Financial situation** (ref. good)																				

poor	0.47	0.21	1.26	0.093		0.39	0.17	1.07	0.043	*	0.85	0.42	1.72	0.644		0.82	0.40	1.68	0.585	

moderate	0.68	0.46	1.02	0.054		0.64	0.43	0.98	0.034	*	0.88	0.68	1.14	0.340		0.84	0.65	1.11	0.218	

**CAMBODIA**																				

	**IN CARE = 1 (AMONG THE DIAGNOSED; N = 925)**										**IN TREATMENT = 1 (AMONG THE LINKED TO CARE; N= 917)**									
	
	**CUDE OR**	**CI-95**		**P-VALUE**		**AOR**	**CI-95**		**P-VALUE**		**CRUDE OR**	**CI-95**		**P-VALUE**			**AOR**	**CI-95**	**P-VALUE**	

**Gender** (ref. men)																				

women	1.62	1.01	2.61	0.046	*	1.80	1.03	3.13	0.036	*	5.42	1.32	22.23	0.019	*	4.38	0.78	24.57	0.093	

**Education** (ref. high (higher secondary or higher))																				

low (primary school or lower)	0.92	0.23	3.61	0.906		1.24	0.35	4.39	0.739		0.83	0.10	6.98	0.869		0.36	0.03	4.18	0.414	

middle (lower secondary school)	0.75	0.16	3.40	0.708		1.01	0.23	4.39	0.985		//	//	//	//		//	//	//	//	

**Financial situation** (ref. good)																				

poor	0.30	0.18	0.49	0.000	***	0.27	0.16	0.46	<0.001	***	1.30	0.21	8.13	0.777		1.42	0.24	8.72	0.683	

moderate	0.35	0.18	0.65	0.001	***	0.37	0.19	0.72	0.003	**	0.38	0.07	1.88	0.240		0.54	0.09	3.01	0.487	

	**ADHERED TO TREATMENT = 1 (AMONG THOSE IN TREATMENT; N = 522)**										**UNDER CONTROL = 1 (AMONG THOSE ADHERED TO CARE; N = 356)**									
	
	**CRUDE OR**	**CI-95**		**P-VALUE**		**AOR**	**CI-95**		**P-VALUE**		**CRUDE OR**	**CI-95**		**P-VALUE**			**AOR**	**CI-95**	**P-VALUE**	

**Gender** (ref. men)																				

women	2.23	1.65	3.00	0.001	***	2.11	1.57	2.84	<0.001											

**Education** (ref. high (higher secondary or higher))																				

low (primary school or lower)	1.11	0.47	2.57	0.810		0.63	0.28	1.34	0.251		1.04	0.39	2.77	0.925		0.52	0.21	1.31	0.166	

middle (lower secondary school)	0.99	0.38	2.58	0.993		0.71	0.28	1.75	0.460		0.91	0.29	2.84	0.870		0.62	0.21	1.82	0.383	

**Financial situation** (ref. good)																				

poor	0.71	0.54	0.92	0.009	**	0.74	0.56	0.99	0.045	*	0.70	0.51	0.95	0.025	*	0.74	0.52	1.04	0.088	

moderate	0.84	0.61	1.16	0.305		0.89	0.62	1.27	0.531		0.99	0.66	1.47	0.972		1.07	0.69	1.64	0.745	


*Notes:* OR = odds ratio; AOR = adjusted odds ratio; adjusted for age, body mass index, current smoking status and comorbidity diabetes type 2; CI = Confidence interval; *p < 0.05 **p < 0.01 ***p < 0.001; ^a^ the BELHES sample is only a subsample of the HIS sample and the sample size was too small for a logistic regression with outcome variable ‘under control’; ^b^ only adjusted for age; // = not able to estimate ORs because of small cells.

Only in Belgium, we found a significant effect of educational level: individuals with a low educational level were less likely to be ‘adherent to care’. Poor financial situation was a significant determinant of HTN care in all three countries, but it was associated with different gaps in the cascade. In Belgium, patients with a poor financial situation were less likely to be ‘in treatment’. In Slovenia, having a poorer financial situation was negatively related to ‘adhering to treatment’. In Cambodia, associations were observed throughout almost the entire cascade: HTN patients with poor or moderate financial status were less likely to be ‘linked to care’ and ‘adherent to treatment’.

### Qualitative reflections on the results

#### ‘Tested’

Belgian experts expected the high number of people tested, as this is part of the ICP element ‘Identification’ (Figure 1), which is the most implemented element in all PC types in Belgium (average[SD]:4.31[0.25]). Also the Cambodian experts referred to the well-implementation of the ‘Identification’ element in all ODs (4.2[0.2]) for the relatively high ‘tested’ bar, in particular among women. Although Slovenian scores were even higher on this ICP element (4.9[0.1]), the bar ‘tested’ was much lower. Slovenian experts suggest three potential explanations, all related to the argument that these low numbers are probably not reflecting the actual number of people tested. First, the Slovenian guideline on HTN screening recommends having a BP measurement at least every 5 years for people aged 30 years or older [35] instead of every 3 years. Second, all employees are required to have a medical check-up by an occupational physician every 1–5 years, including measuring BP (and these checks are not included in the data), thus, they probably –especially when their test was okay– would no longer seek for another check-up by the practice nurse. Third, and probably the most important explanation, is the fact that since 2011, a countrywide treatment protocol [43] has been implemented including a HTN screening program by a practice nurse; however, a lot of people still go directly to their family doctors, who probably register BP measurements less systematically and, even if they do, there is no systematic interface with the CHCL health information system, and thus are not available for analysis.

#### ‘Diagnosed’

The Belgian experts found the gap between ‘testing and diagnosis in people with elevated blood pressure striking, especially as Cambodia has similar diagnosis rates (even higher among women) with a much lower testing rate.

Several potential explanations emerged during the Belgian FGD. First, methodological issues may play a role. As the ‘diagnosed’ bar is based on self-reported high BP, patients who take antihypertensive medication and have achieved BP control may report they no longer have HTN (simplified as “high BP”). Also, the prevalence is estimated on a subsample of the HIS (the Belgium EHES) while the diagnosed bar is estimated on the HIS. In neighbouring countries, such as Luxemburg, using similar data (EHES LUX) and the same way of operationalization, similar figures were found [44]. Second, the experts refer to diagnostic inertia (i.e. when physicians observe elevated BP measurements but label the respective patients as normotensive [45]) as potential explanation: GPs probably also take other cardiovascular risk factors into account before diagnosing, or they consider high BP as one of the many health problems of the patient, and therefore prioritizing it less and not explicitly labelling it as a HTN diagnosis. GPs may also prefer to prescribe first a 24h BP measurement or a self-measurement of BP during a week to be able to make a diagnosis with more certainty. Last, experts argue that it is also possible that people take antihypertensive medication without knowing the indication. Experts linked this to the low implementation of the ‘health education’ (0.62[0.84]) and ‘self-management support’ (2.01[0.59]) elements.

#### ‘In care’ and ‘in treatment’

The Belgian experts related the high ‘in care’ and ‘in treatment’ bar to the relatively high level of implementation of the ‘treatment’ ICP element (3.65[0.38]) and the overall strong health care system. Although the implementation of the ‘treatment’ ICP element in Slovenia is even higher, this was not reflected in higher ‘in care’ and ‘in treatment’ bars. Experts of Slovenia refer again to the possibility that a lot of patients go directly to their family doctor instead of having the protocol-prescribed yearly HTN consult with the practice nurse. Thus, this gap rather reflects the poor referral of newly diagnosed patients to care performed by nurse practitioner. Cambodian experts were surprised about the relatively high ‘in care’ and ‘in treatment’ bars, because the’ treatment’ ICP element was poorly implemented (2.4[0.17]). They also refer to the strong overlap in operationalization of the ’in care’ and ‘in treatment’ bar to explain the almost non-existent gap.

#### ‘Adherent to treatment’ and ‘under control’

Slovenian experts accentuate that once patients are registered in HTN care by the nurse, the attrition through the cascade is smaller than in Belgium and especially than in Cambodia. They attribute this to their strong PC system and the high level of implementation of integrated care (3.72[1.21]), with a very important role of the nurse practitioner. Cambodian experts, in contrast, highlight that retaining patients in care and treatment is a larger challenge in the Cambodian HC system, as reflected in the large gap between ‘treatment adherence’ and ‘under control’. Together with the Belgian experts, they argue that for better continuum and outcomes of care, a higher overall implementation of integrated care may play an important role.

#### Gender differences

Slovenian experts were puzzled by the gender difference in ‘testing’, as women are often found to be more involved with preventive care. They refer again to the medical check-ups as potential explanation, as more men are employed, and especially in sectors where these check-ups are more regularly required. If their blood pressure was too high during the occupational check-up, they were referred to a primary care practice for a re-check, likely resulting in higher testing rates among men. However, once diagnosed, the gender difference is small and reversed in Slovenia. Cambodian experts understood the gender differences, with women having less attrition throughout the cascade, in light of dominant traditional gender roles, with men generating the income for the family, and therefore having less time to go to the HC facility. This is in line with the results of other health prevention campaigns. Women have more contact with health services, mainly due to maternal care and care for their children.

#### Socioeconomic vulnerability

Belgian experts linked the positive relation between education and being ‘adherent to care’ to the weak implementation of ‘health education’ and ‘self-management support’ in the Belgium PC system, which could also very well apply in Slovenia and more so in Cambodia. Although Belgium is a very rich country with a strong PC orientation, its PC system scores ‘weak’ on the ‘accessibility‘ dimension of Kringos [24,25] (S1.b). Experts recognize that, although Belgium has done a lot of efforts to increase the financial accessibility of HC (e.g. the increased reimbursement status, maximum bill) [46] the direct costs —for instance [47]— in combination with the indirect costs of HTN treatment (GP consultation, transport costs, special diet, etc.) may be still too high for the most economically-vulnerable groups, explaining their lower changes to be ‘in treatment’. They also indicate that because the HTN symptoms are not directly tangible, it may be less prioritized, particularly among vulnerable patients who probably have multiple problems. This explanation is echoed by the Slovenian experts for the lower ‘adherent to treatment’ among economic vulnerable patients, as they did not consider the cost of HTN treatment as a barrier in itself. They emphasized the strong accessibility of the Slovenian PC system, which is also recognized in the work of Kringos [24,25]. Cambodian experts attribute the inequalities in HTN care by income to the high out-of-pocket payments, the limited human and financial resources within public HC and the fact that private HC and medications are quite expensive.

## Discussion

In this mixed methods study, we estimated three HTN CoCs, using survey data for Belgium and Cambodia and register data for Slovenia. Important lessons were learnt by comparing the CoCs between different HC systems with varying degrees of ICP implementation. We found that, ultimately in all three CoCs, less than half of those diagnosed achieved well-controlled HTN, and socioeconomic vulnerability was a key determinant of dropout across the continuum of care. However, also substantial differences were observed between the three HC systems with respect to regarding where most patients were lost along the continuum of care, the magnitude of these losses and the extent to which the cascades were gender-stratified.

In Belgium, the largest gap is between ‘prevalence’ and ‘diagnosis’; once diagnosed, the health system retains patients fairly well along the continuum of care until ‘treatment adherence’. In Cambodia, a large drop –especially among men– is found at the beginning of the care continuum (‘testing’), and at the end of the cascade, between ‘treatment adherence’ and ‘under control’. In Slovenia, the largest attrition is at the beginning of the cascade, with only a limited number of patients tested and linked to care. However, once registered in HTN care, the losses through the cascade are limited.

The high implementation of the ICP components ‘identification’ and ‘treatment’ has been linked to the high testing rates and minimal dropout between ‘in care’ and ‘in treatment’ in Belgium. Cambodian experts refer to the overall low degree of ICP implementation and limited financial and human resources for explaining the large gaps between ‘in treatment’, ‘treatment adherence’ and ‘under control’. Although previous research found higher BP control rates in integrated care models [9,10,11], this was not reflected in our results. While the overall level of ICP implementation was highest in Slovenia, the cascade attainment in Belgium was better. However, the Slovenian cascade needs careful consideration, as experts identified that some gaps (low testing rates, large gap between ‘diagnosed’ and ‘in care’) reflect limitations of the health information system rather than ‘real’ gaps in the care continuum.

Given that this is a real-world study rather than a randomized controlled trial, broader contextual and HC system factors variables, may also play a critical role. Cross-country research among low- and middle-income countries [48] also found that higher GDP per capita correlates with better performance at each step of the cascade. While Belgium and Slovenia are both high income countries, the GDP per capita is higher in Belgium. Belgium’s PC system also scores high on the dimension continuity and comprehensiveness, which were already stressed as important for HTN management [48], while these dimensions are weak in the Slovenian PC system.

The availability and affordability of HTN medication and out-of-pocket costs may also have an important impact on HTN treatment and control, and differences in the stratification of the CoC according to patients SES [49]. In addition, research showed that adherence to treatment declines as out-of-pocket expenditure increases [50]. In Cambodia, high out-of-pocket cost, financial and human resources constraints, and a mis-match between the demand and supply of antihypertensives [51], may contribute to the lower ‘treatment adherence’ and ‘under control’ bars, and the consistent social gradient observed in all steps of the cascade, with the least well-off being more likely to dropout of HTN care.

Similarly, in the HICs Belgium and Slovenia, socioeconomically vulnerable patients have a higher likelihood to be lost through the cascade at least in one of the steps. In Belgium, HTN patients with a poor financial situation were less likely to be ‘in treatment’; those with a low education level in Belgium and a poor financial situation in Slovenia were less likely to be ‘adherent to treatment’. These results, as explained by the experts, show that, for the improvement of HTN management at population level with particular attention to socioeconomic vulnerable patients, strategies beyond access and affordability of care are also required [49,52]. It is also a call for more investments in health literacy and self-management skills —important capability factors of HTN management [52]—which are often worse among socioeconomic vulnerable patients [53].

In Cambodia and especially in Belgium, there is almost no implementation of the ICP grid element ‘health education’; all three countries are underperforming in terms of self-management support by HC providers. In light of limited resources in Cambodia, one potential pathway to produce more equitable access to essential HTN care is the double strategy of building additional community-based care (to improve access) while improving the quality of facility-based care (to improve health outcomes) [27]. For Belgium, the integration of nurses in practice should be further stimulated by govermental support and funding. A Belgium study [31] has already shown that practices with a nurse have a higher ICP implementation, especialy regarding the dimensions self-management support and health education. In the majority of Random Controlled Trails, nurse-led care was also associated with improved blood pressure control [54]. In Slovenia, a strong facilitator for the implementation of health education was the reorganization of family medicine teams by integration of a nurse practitioner and the use of peer supporters [55].

As a final key finding, we highlight the differences in the extent of gender stratification in the HTN CoC between the two high-income countries and Cambodia. Gender equality at the macro level differs markedly between Belgium and Slovenia, on the one hand, and Cambodia, on the other. Belgium and Slovenia rank high on the Gender Equality Index, particularly in the health domain, and low on the Gender Inequality Index (see S1c), which may explain the minimal (Slovenia) or absent (Belgium) gender differences observed within their HTN CoCs. In contrast, Cambodia has a higher Gender Inequality Index score and remains strongly influenced by traditional gender norms. These norms seem to contribute to lower dropout rates for women throughout the HTN CoC. This gender-stratified CoC aligns with findings from other low- and middle-income countries [56], likely due in part to traditional caregiving roles and social norms that emphasize women’s responsibility for family health, including their own health.

There are also some limitations that should be addressed. We were not able to link the ICP Grid scores with the CoCs in a statistical way, but we highlight the strength of complementing quantitative data with qualitative data to interpret the CoC in the light of countries’ degree of integrated care. This combined approach was crucial for reaching –as much as possible– comparable bars and determinants of the CoC. Comparisons between the three countries could not be made in a straightforward way, as they need to be contextualized and reviewed in terms of differences in the data sources and health information systems, which have their own limitations. Furthermore, the survey data used for Belgian and Cambodian CoC are subject to recall bias and reporting social desirable answers [57]. The quality of administrative data from Electronic Health Record (EHRs)–used for the Slovenian CoC– depends strongly on the registration quality of health providers [58]. This makes it difficult to distinguish gaps in the care continuum from gaps in the health information system. Another limitation is that the data only cover patients who are already in the health services and have an EHR. As a result, there is no information about people who are undiagnosed and thus no reliable prevalence of HTN can be estimated.

## Conclusion

The cascades of HTN care were built differently across the three countries, and thus direct comparisons would not be reasonable. However, in identifying the reasons behind the performance of each country’s CoC and linking this with the HTN ICP, we deduce several lessons. Belgium and Slovenia both have, principally, a primary care-oriented approach and could take inspiration from Cambodia on their broader primary healthcare approach that involves community-based care in the form of peer supporters and community-based healthcare workers. This could address the large gap between ‘prevalence’ and ‘diagnosis’ in Belgium and the gap between ‘diagnosed’ and ‘in care’ in Slovenia; as well as to augment health education and self-management support provision. To note, Slovenia has started making use of nurse-led care for chronic conditions, which Belgium can emulate to shift tasks from an overburdened cadre of GPs. Cambodia can learn on retention of patients in care and how Belgium scores high on continuity of care. We expect countries with similar contexts to also learn from this exercise.

## Additional Files

The additional files for this article can be found as follows:

10.5334/ijic.8921.s1 S1.a. Characteristics of the health care system and its primary health care orientation based on Reibling’s typology.

10.5334/ijic.8921.s2 S1.b. The strengths of countries’ primary care dimensions based on Kringos’ scoring system.

10.5334/ijic.8921.s3 S1.c. Socioeconomic and demographic characteristics and health indicators of Belgium, Slovenia and Cambodia.

10.5334/ijic.8921.s4 S2. Operationalization of the potential determinants of the gaps in the hypertension cascade of care.

10.5334/ijic.8921.s5 S3. Sampling information of the ICP GRID data per country.

10.5334/ijic.8921.s6 S4.a. The ICP Grid scores of Belgium.

10.5334/ijic.8921.s7 S4.b. The ICP Grid scores of Slovenia.

10.5334/ijic.8921.s8 S4.c. The ICP Grid scores of Cambodia.

10.5334/ijic.8921.s9 S5. Information about the Focus Group Discussions and the respondents.

10.5334/ijic.8921.s10 S6. Country and gender specific HTN cascade.

10.5334/ijic.8921.s11 S7. Description of the determinants in the sample of the diagnosed HTN patients.

## Abbreviations

HTN – Hypertension

HC – Health Care

PC – Primary Care

FLHS – First Line Health System

ICP – Integrated Care Package

CoC – Cascade of Care

GP – General Practitioner

EHR – Electronic Health Record

## References

[1] Mills KT, Stefanescu A, He J. The global epidemiology of hypertension. Nature Reviews Nephrology. 2020;16(4):223–237. DOI: 10.1038/s41581-019-0244-2 32024986 PMC7998524

[2] Stanaway JD, et al. Global, regional, and national comparative risk assessment of 84 behavioural, environmental and occupational, and metabolic risks or clusters of risks for 195 countries and territories, 1990–2017: a systematic analysis for the Global Burden of Disease Study 2017. Lancet. 2018;392(10159):1923–1994. 30496105 10.1016/S0140-6736(18)32225-6 PMC6227755

[3] Marra C, et al. Cost-effectiveness of pharmacist care for managing hypertension in Canada. Canadian Pharmacists Journal. 2017;150(3):184–197. DOI: 10.1177/1715163517701109 28507654 PMC5415065

[4] Dugani S, Gaziano TA. 25 by 25: Achieving Global Reduction in Cardiovascular Mortality. Current Cardiology Reports. 2016;18(1). DOI: 10.1007/s11886-015-0679-4 PMC4797715 26748650

[5] Mills KT, et al. Global Disparities of Hypertension Prevalence and Control A Systematic Analysis of Population-Based Studies From 90 Countries. Circulation. 2016;134(6):441–450. DOI: 10.1161/CIRCULATIONAHA.115.018912 27502908 PMC4979614

[6] Santosa A, Zhang Y, Weinehall L, et al. Gender differences and determinants of prevalence, awareness, treatment and control of hypertension among adults in China and Sweden. BMC Public Health. 2020;1763. DOI: 10.1186/s12889-020-09862-4 33228600 PMC7685617

[7] Connelly PJ, Currie G, Delles C. Sex Differences in the Prevalence, Outcomes and Management of Hypertension. Curr Hypertens Rep. 2022;24:185–192. DOI: 10.1007/s11906-022-01183-8 35254589 PMC9239955

[8] Mohanty SK, et al. Awareness, treatment, and control of hypertension in adults aged 45 years and over and their spouses in India: A nationally representative cross-sectional study. Plos Medicine. 2021;18(8):e1003740. DOI: 10.1371/journal.pmed.1003740 34428221 PMC8425529

[9] Chow CK, et al. Prevalence, Awareness, Treatment, and Control of Hypertension in Rural and Urban Communities in High-, Middle-, and Low-Income Countries. Jama-Journal of the American Medical Association. 2013;310(9):959–968. DOI: 10.1001/jama.2013.184182 24002282

[10] Peters MA, et al. Evidence for an expanded hypertension care cascade in low- and middle-income countries: a scoping review. Bmc Health Services Research. 2022;22(827). DOI: 10.1186/s12913-022-08190-0 PMC9235242 35761254

[11] Zhang YT, et al. Effects of integrated chronic care models on hypertension outcomes and spending: a multi-town clustered randomized trial in China. Bmc Public Health. 2017;17(244). DOI: 10.1186/s12889-017-4141-y PMC5346199 28284202

[12] Glynn LG, et al. Interventions used to improve control of blood pressure in patients with hypertension. Cochrane Database of Systematic Reviews. 2010;17(3):CD005182. DOI: 10.1002/14651858.CD005182.pub4 PMC13248079 20238338

[13] Jaffe MG, et al. Improved Blood Pressure Control Associated With a Large-Scale Hypertension Program. Jama-Journal of the American Medical Association. 2013;310(7):699–705. DOI: 10.1001/jama.2013.108769 PMC4270203 23989679

[14] Raak A, et al. Integrated care in Europe. Description and comparison of integrated care in six EU countries. Maarssen: Elsevier Gezondheidzorg. 208; 2003.

[15] WHO. Implementation Tools: Package of Essential Noncommunicable (PEN) Disease Interventions for Primary Health Care in Low-Resource Settings. Geneva, Switzerland: WHO; 2013.

[16] Demaio AR, et al. Primary Health Care: a strategic framework for the prevention and control of chronic non-communicable disease. Global Health Action. 2014;7(24504). DOI: 10.3402/gha.v7.24504 PMC4122819 25095779

[17] Stojnić N, et al. Evaluation of the Implementation of Integrated Primary Care for Patients with Type 2 Diabetes and Hypertension in Belgium, Cambodia, and Slovenia. International Journal of Integrated Care. 2024;24(2):27,1–12. DOI: 10.5334/ijic.7664 38948162 PMC11212773

[18] Egan BM, Zhao YM, Axon RN. US Trends in Prevalence, Awareness, Treatment, and Control of Hypertension, 1988–2008. Jama-Journal of the American Medical Association. 2010;303(20):2043–2050. DOI: 10.1001/jama.2010.650 20501926

[19] Wozniak G, et al. Hypertension Control Cascade: A Framework to Improve Hypertension Awareness, Treatment, and Control. J Clin Hypertens (Greenwich). 2016;18(3):232–239. DOI: 10.1111/jch.12654 PMC5049660 26337797

[20] Menendez E, et al. Prevalence, Diagnosis, Treatment, and Control of Hypertension in Spain. Results of the Di@bet.es Study. Revista Espanola De Cardiologia. 2016;69(6):572–578. DOI: 10.1016/j.rec.2015.11.034 26979767

[21] Ikeda N, et al. Control of hypertension with medication: a comparative analysis of national surveys in 20 countries. Bulletin of the World Health Organization. 2014;92(1):10–19. DOI: 10.2471/BLT.13.121954 24391296 PMC3865548

[22] van Olmen J, et al. Scale-up integrated care for diabetes and hypertension in Cambodia, Slovenia and Belgium (SCUBY): a study design for a quasi-experimental multiple case study. Global Health Action. 2020;13(1):1824382. DOI: 10.1080/16549716.2020.1824382 33373278 PMC7594757

[23] Reibling N, Ariaans M, Wendt C. Worlds of Healthcare: A Healthcare System Typology of OECD Countries. Health Policy. 2019;123(7):611–620. DOI: 10.1016/j.healthpol.2019.05.001 31133444

[24] Kringos D, et al. The strength of primary care in Europe: an international comparative study. British Journal of General Practice. 2013;63(616):E742–E750. DOI: 10.3399/bjgp13X674422 PMC3809427 24267857

[25] Lember M, Cartier T, Bourgueil Y. Chapter 2: Structure and organization of primary care. In: Kringos DS, et al. Editors. Building primary care in a changing Europe. Observatory Study Series WHO; 2015.

[26] THE THIRD HEALTH STRATEGIC PLAN 2016–2020 (HSP3) “Quality, Effective and Equitable Health Services”. 2016, Department of Planning and Health Information, Ministry of Health.: Cambodia.

[27] Chham S, et al. The cascade of hypertension care in Cambodia: evidence from a cross-sectional population-based survey. Bmc Health Services Research. 2022;22(1). DOI: 10.1186/s12913-022-08232-7 PMC9241312 35768805

[28] Bos P, Wouters E, Danhieux K. et al. Unravelling the Belgian cascade of hypertension care and its determinants: insights from a cross-sectional analysis. BMC Public Health. 2024;24:1559. DOI: 10.1186/s12889-024-19010-x 38872180 PMC11177511

[29] Te V, Long S, Damme WV, Ir P, Wouters E, van Olmen J. An in-depth Analysis of the Degree of Implementation of Integrated Care for Diabetes in Primary Health Care in Cambodia. International Journal of Integrated Care. 2024;24(4):11,1–10. DOI: 10.5334/ijic.7602 PMC11623098 39650248

[30] Klemenc-Ketis Z, et al. Implementation of integrated primary care for patients with diabetes and hypertension: a case from Slovenia. International Journal of Integrated Care. 2021;21(3). DOI: 10.5334/ijic.5637 PMC8485865 34690619

[31] Danhieux K, Buffel V, Remmen R, et al. Scale-up of a chronic care model-based programme for type 2 diabetes in Belgium: a mixed-methods study. BMC Health Serv Res. 2023;23:141. DOI: 10.1186/s12913-023-09115-1 36759890 PMC9911183

[32] Creswell JW, Creswell JD. Mixed methods procedures. In: Research design: Qualitative, quantitative, and mixed methods approaches (5th ed.). 2018; pp. 213–246. Los Angeles, CA: SAGE Publications, Inc.

[33] Zhou B, et al. Long-term and recent trends in hypertension awareness, treatment, and control in 12 high-income countries: an analysis of 123 nationally representative surveys. The Lancet. 2019;394(10199):639–651. DOI: 10.1016/S0140-6736(19)31145-6 PMC6717084 31327564

[34] de Cort P, et al. Aanbeveling voor goede medische praktijkvoering: hypertensie (herziening). Huisarts nu: maandblad van de Wetenschappelijke Vereniging van Vlaamse Huisartsen. Brussel. 2009;38(9):340–361.

[35] Blinc A, et al. 2018. Ljubljana: Združenje za arterijsko hipertenzijo, Slovensko zdravniško društvo; 2019. https://hipertenzija.org/wp-content/uploads/2019/12/Smernice-za-obravnavo-hipertenzije-2018.pdf

[36] Medicine DoP. National standard operating procedure for diabetes and hypertension management in primary care 2019. Cambodia: Ministry of Health; 2019.

[37] Demarest S, Berete F, Charafeddine S, Van der Heyden J. Gezondheidsenquête. 2018: Methodologie. Brussel, België: Sciensano. Rapportnummer: D/2019/14.440/34. Accessible: www.gezondheidsenquete.be

[38] Nguyen D, et al. The Belgian health examination survey: objectives, design and methods. Archives of Public Health. 2020;78(1). DOI: 10.1186/s13690-020-00428-9 PMC7268416 32514346

[39] Te V, et al. Generation of cascades of care for diabetes and hypertension care continuum in Cambodia: protocol for a population-based survey protocol. JMIR Res Protoc. 2022;11(9):e36747. DOI: 10.2196/36747 36053576 PMC9482065

[40] Bowskill R, Clatworthy J, Parham R, Rank T, Horne R. Patients’ perceptions of information received about medication prescribed for bipolar disorder: implications for informed choice. J Affect Disord. 2007;100(1–3):253–257. DOI: 10.1016/j.jad.2006.10.018 17174406

[41] Mårdby A-C, Åkerlind I, Jörgensen T. Beliefs about medicines and self-reported adherence among pharmacy clients. Patient Educ Couns. 2007;69(1):158–164. DOI: 10.1016/j.pec.2007.08.011 17913439

[42] World Population Prospects: The 2010 Revision, Volume I: Comprehensive Tables. United Nations, Department of Economic and Social Affairs, Population Division; 2011.

[43] Klemenc-Ketiš Z, Švab I, Poplas Susič A. Implementing Quality Indicators for Diabetes and Hypertension in Family Medicine in Slovenia. Zdr Varst. 2017;56(4):211–219. DOI: 10.1515/sjph-2017-0029 29062395 PMC5639810

[44] Bocquet V, Barré J, Couffignal S, et al. Study design and characteristics of the Luxembourg European Health Examination Survey (EHES-LUX). BMC Public Health. 2018;18(1169). DOI: 10.1186/s12889-018-6087-0 PMC6182799 30309333

[45] De Backer T, Van Nieuwenhuyse B, De Bacquer D. Antihypertensive treatment in a general uncontrolled hypertensive population in Belgium and Luxembourg in primary care: Therapeutic inertia and treatment simplification. The SIMPLIFY study. PLoS One. 2021;16(4):e0248471. DOI: 10.1371/journal.pone.0248471 33819268 PMC8021160

[46] Gerkens S, Merkur S. Belgium: health system review. Health systems in transition. 2020;1–237. 33527904

[47] Karakaya G, Umbach I. Analyse van de behandelingen met antihypertensiva [Analysis of antihypertensive treatments]. Brussel, België: Onafhankelijke Ziekenfondsen; 2023.

[48] Geldsetzer P, et al. The state of hypertension care in 44 low-income and middle-income countries: a cross-sectional study of nationally representative individual-level data from 1.1 million adults. Lancet. 2019;394(10199):652–662. DOI: 10.1016/S0140-6736(19)30955-9 31327566

[49] Attaei MW, et al. Availability and affordability of blood pressure-lowering medicines and the effect on blood pressure control in high-income, middle-income, and low-income countries: an analysis of the PURE study data. Lancet Public Health. 2017;2(9):E411–E419. 29253412 10.1016/S2468-2667(17)30141-X

[50] Viswanathan M, et al. Interventions to Improve Adherence to Self-administered Medications for Chronic Diseases in the United States A Systematic Review. Annals of Internal Medicine. 2012;157(11):785–795. DOI: 10.7326/0003-4819-157-11-201212040-00538 22964778

[51] Chham S, et al. Scaling-up integrated type-2 diabetes and hypertension care in Cambodia: what are the barriers to health system performance? Frontiers in Public Health. 2023;11(1136520). DOI: 10.3389/fpubh.2023.1136520 PMC10272385 37333565

[52] Khatib R, et al. Patient and Healthcare Provider Barriers to Hypertension Awareness, Treatment and Follow Up: A Systematic Review and Meta-Analysis of Qualitative and Quantitative Studies. Plos One. 2014;9(1). DOI: 10.1371/journal.pone.0084238 PMC3893097 24454721

[53] Woodward A, et al. Self-management of multiple long-term conditions: A systematic review of the barriers and facilitators amongst people experiencing socioeconomic deprivation. PLoS One. 2023;12(2):e0282036. DOI: 10.1371/journal.pone.0282036 PMC9942951 36809286

[54] Stephen C, et al. Nurse-led interventions to manage hypertension in general practice: A systematic review and meta-analysis. J Adv Nurs. 2022;78(5):1281–1293. DOI: 10.1111/jan.15159 35244944

[55] Virtič T, et al. Peer Support as Part of Scaling-Up Integrated Care in Patients with Type 2 Diabetes and Arterial Hypertension at the Primary Healthcare Level: A Study Protocol. Zdr Varst. 2023;62(2):93–100. DOI: 10.2478/sjph-2023-0013 37266071 PMC10231890

[56] Irazola VE, et al. Hypertension Prevalence, Awareness, Treatment, and Control in Selected LMIC Communities: Results From the NHLBI/UHG Network of Centers of Excellence for Chronic Diseases. Glob Heart. 2016;11(1):47–59. DOI: 10.1016/j.gheart.2015.12.008 27102022 PMC4843831

[57] Atwood KM, et al. Comparison of Diagnosed, Self-Reported, and Physically-Measured Hypertension in Canada. Canadian Journal of Cardiology. 2013;29(5):606–612. DOI: 10.1016/j.cjca.2012.11.019 23395221

[58] Leong A, et al. Systematic Review and Meta-Analysis of Validation Studies on a Diabetes Case Definition from Health Administrative Records. Plos One. 2013;8(10). DOI: 10.1371/journal.pone.0075256 PMC3793995 24130696

[59] Bonomi AE, et al. Assessment of Chronic Illness Care (ACIC): A practical tool to measure quality improvement. Health Services Research. 2002;37(3):791–820. DOI: 10.1111/1475-6773.00049 12132606 PMC1434662

