# Supplementary material for: Hypertension Cascade Across Three Healthcare Systems and in Relation to the Level of Implementation of the Integrated Care Package
Source: Int J Integr Care. 2025 Aug 22;25(3):22. doi: 10.5334/ijic.8921 (PMC12372687; doi:10.5334/ijic.8921)
Supplement: S1.a. — Characteristics of the health care system and its primary health care orientation based on Reibling’s typology. [file ijic-25-3-8921-s1.pdf]

S1.a. Characteristics of the health care system and its primary health care orientation based on Reibling's typology

	Country	indicator	Belgium	Slovenia	Cambodia
HC	supply	**Health expenditure** per capita in US$ PPP[(a)]	5458.4	3303.5	113
		Health expenditure, as % of GDP [(a)]	8.2%	7.3%	6.9%
		**Number of GPs** per 1000 inhabitants[(1)]	1.12	0.57	1.8[(b)]
		Number of Physicians per 1000 inhabitants [(a)]	6.0	3.2 (2018)	0.2 (2014)
		Number of Nurses and midwives per 1000 inhabitants [(a)]	11.8 (2015)	10.2 (2018)	1.0
	Public-Private Mix	**Public health expenditure,** % of total health expenditure	77.37	71.78	22.3[(c)]
		Domestic general government health expenditure, % of total health expenditure (a)	76.78	72.37	24.31
		Domestic private government health expenditure, % of total health expenditure (a)	23.22	27.63	69.19
		External health expenditure, % of total health expenditure (a)	/	/	6.5
		Private household **out-of-pocket expenditure**, % of total expenditure[(a)]	18.17	11.66	64.39
		**Remuneration of specialists** (0 = fee-for-service, 1= salary)	0	0	0 / 1 [(d)(2)]
	Access Regulation	**Access Regulation Index** (0-3)	0	3	0
		(a) GP registration (0=no, 1=yes)	0 [(3)]	1	0
		(b) Specialist access (2= Referral, 1 = Co-payment, 0 = Free)	0	2	0
		**Choice** (1 = if choice is limited in one sector, 0 = otherwise)	0	0	0
		**Cost Sharing for GP visits** (1 = Yes, 0 = No)	1	1	0
		**Choice of GP** (1 = Limited, 0 = Free)	0	0	0
		**Choice of specialist** (1 = Limited, 0 = Free)	0	0	0
		**Choice of hospital** (1 = Limited, 0 = Free)	0	0	0
PC	Primary care orientation	Health expenditure on **outpatient care** (EU min: 17; max: 49)	18.3	24.5	
		Spending on primary health care services as share of current health expenditure	12 (2020)[)(e)]	17 (2020)[(e)]	67 [(f)]
		Ratio of **general practitioners/ specialists** (EU: min: 0.14; max: 1.47)	0.62	0.29	3.98 (2743/689)[(d)]

Notes: *By default the numbers are of 2019, (1) For Belgium and Slovenia the initial information in Reibling's work was based on OECD Health Data 2016 (countries' average values for the years 2011 to 2014) and Countries' Health in Transition Report (HiT) provided by the European Observatory on Health Systems and Policies (with 2013 as reference year). We have updated these numbers to 2019 (or with the most available data), and extended by some specification based on data of the Woldbank.org. For Cambodia: the official tracking indicator for health human resource is the number of physician/nurse/midwives per 1000 inhabitants, (2) The remuneration of specialist in Cambodia is based on the health facility they serve. If they are civil servant, they have a salary, if they are self-employed, it is a fee-for-service system, (3) BE: only in GP practices with a capitation system patients need to be inscribed (but this system covers only 4.17% of the Patients in 2019 (based on the IMA-atlas https://atlas.ima-aim.be/databanken/?rw=1&lang=nl)*

Sources: *(a) Worldbank.org; (b) National Health Account 2012-2016; (c) Annual Health Progress Report 2018; (d) The Kingdom of Cambodia: Health System review, 2015 Pacific Observatory on Health Systems and Policies, World Health, health systems in transition); (d) Worldbank.org; (e) https://www.oecd-ilibrary.org/social-issues-migration-health/spending-on-primary-health-care-services-as-share-of-current-health-expenditure-2020_9c97d03e-en (f) https://www.who.int/cambodia/news/commentaries/detail/primary-healthcare-remains-the-foundation-for-all-in-cambodia*
