# Supplementary material for: Hypertension Cascade Across Three Healthcare Systems and in Relation to the Level of Implementation of the Integrated Care Package
Source: Int J Integr Care. 2025 Aug 22;25(3):22. doi: 10.5334/ijic.8921 (PMC12372687; doi:10.5334/ijic.8921)
Supplement: S1.b. — The strengths of countries’ primary care dimensions based on Kringos’ scoring system. [file ijic-25-3-8921-s2.pdf]

S1.b. The strengths of countries' primary care dimensions based on Kringos' scoring system

	Structure of PC			Service delivery process				
country	PC governance	Economic conditions of PC	PC workforce development	Access to PC	Continuity of PC	Coordination of PC	Comprehensiveness of PC	Overal PC system strenght
Belgium	Medium	Strong	Medium	Weak	Strong	Medium	Strong	Strong
Slovenia	Strong	Strong	Strong	Strong	Weak	Strong	Weak	strong
Cambodia								

***Note**: for more information about the dimensions, please see the work of Kringos [24-25]; for Cambodia there is no information, as this country was not included in the project of Kringos and colleagues.*
