# Supplementary material for: Hypertension Cascade Across Three Healthcare Systems and in Relation to the Level of Implementation of the Integrated Care Package
Source: Int J Integr Care. 2025 Aug 22;25(3):22. doi: 10.5334/ijic.8921 (PMC12372687; doi:10.5334/ijic.8921)
Supplement: S1.c. — Socioeconomic and demographic characteristics and health indicators of Belgium, Slovenia and Cambodia. [file ijic-25-3-8921-s3.pdf]

S1.c. Socioeconomic and -demographic characteristics, inequality measures and health indicators of Belgium, Slovenia and Cambodia

**Indicator**	**Belgium**	**Slovenia**	**Cambodia**
Number of inhabitants	11.3 million	2 million	15 million
Country wealth	High income	High income	Low middle income
GDP per capita (US dollar)	46 591	25 943	1 643.12
GDP growth rate	2.1	3.3	7.1
Income inequality (Gini)	0.262	0.246	0.308 (2012)
Gender equality index (1-100)<br>Health domain<br>Gender inequality index (0-1)	76.0 (2023)<br>88.5 (2023)<br>0.044 (2022)	69.4 (2023)<br>86.5 (2023)<br>0.049 (2022)	/<br><br>0.486 (2022)
Poverty rate	14.8	12.0	17.8
Age structure % >= 65	19.01	20.19	4.72
Life expectancy	81.8	81.2 (2018)	69.72

Note: *Numbers of 2019 by default*
Sources*: OECD data; World bank data; European Institute for Gender Equality and Human development data*
