# Supplementary material for: Hypertension Cascade Across Three Healthcare Systems and in Relation to the Level of Implementation of the Integrated Care Package
Source: Int J Integr Care. 2025 Aug 22;25(3):22. doi: 10.5334/ijic.8921 (PMC12372687; doi:10.5334/ijic.8921)
Supplement: S2. — Operationalization of the potential determinants of the gaps in the hypertension cascade of care. [file ijic-25-3-8921-s4.pdf]

S2. Operationalization of the potential determinants of the gaps in the hypertension cascade of care

	**Belgium**	**Slovenia**	**Cambodia**
**Sociodemographics**			
**Age**	40-59 (ref.), 60-69, 70-79		
**Gender**	Men (ref.), Women		
**Socioeconomic vulnerability**			
Low educational level (<= completed primary school)	*Highest level of education:* (Low) primary school or lower; (middle) lower secondary education; (high) higher secondary education or 'higher education' (ref.)	*The last school completed:* (Low) 'unfinished primary school', 'primary school', (Middle) '2- or 3-year vocational school', (High) '4-year high school or gymnasium ', 'college, academy', 'postgraduate study' (ref.)	*Highest educational level:* Low: 'no formal schooling', 'less than primary school', 'primary school' (low) Middle: 'secondary school', 'high school' High: 'college/university', 'post graduate degree' (ref.)
Poor financial situation (vs. good/moderate)	*Able to make ends meet:* (poor) 'with great difficulty', 'with difficulty', 'with some difficulty', (moderate) 'fairly easily', (good) 'easily', 'very easily' (ref.)	*How do you (or your household) cope with the month, taking into account the income you have, what you generate from your own work as well as mutual help between relatives / neighbors / friends?:* (good) 'I am doing well throughout the month' (ref.), (moderate) 'I have problems from time to time', (poor) ' I have problems'	*Based on PCA[a] of household assets:* (Poor) 'Poorest', 'poor', (moderate) 'medium', (rich) 'rich', 'richest' (ref.)
**Life style and health factors**			
BMI	Kg/m2: <25; 25-29; 30+ *HIS: self-reported body weight and length*	Kg/m^2: <25; 25-29; 30+ *measured by a nurse*	Kg/m^2 : <25; 25-29; 30+ *Measured by trained data collector (using Seca)*
Smoking status	*Do you smoke nowadays?:* (yes) 'yes, daily' 'yes, occasionally' vs. (no) 'No, not at all' (ref.)	*Do you smoke?* (yes) 'I am currently smoking' vs. (no) 'I am a former smoker', 'I am exposed to passive smoke', 'I have never smoked' (ref.)	*Do you currently smoke any tobacco products, such as cigarettes, cigars or pipes?* yes vs. no (ref.)
Comorbidity T2D	Self-reported: T2D	Registered diagnose of T2D	Self-reported diagnosis as having been told by healthcare professionals: T2D

***Notes**: [a]PCA: principle component analysis: Ref.= reference category*
