# Supplementary material for: Hypertension Cascade Across Three Healthcare Systems and in Relation to the Level of Implementation of the Integrated Care Package
Source: Int J Integr Care. 2025 Aug 22;25(3):22. doi: 10.5334/ijic.8921 (PMC12372687; doi:10.5334/ijic.8921)
Supplement: S3. — Sampling information of the ICP GRID data per country. [file ijic-25-3-8921-s5.pdf]

S3. Sampling information of the ICP GRID data per country

	**Belgium**	**Slovenia**	**Cambodia**
**Sampling strategy** (purposive)	Stratified by primary care type *(multidisciplinary fee-for-service, monodisciplinary capitation-based practices)* and region *(two urban regions: Ghent and Antwerp; and one rural region: Kempen)*	Stratified by urban/rural *(eight urban health centres in Central Slovenia, and two rural health centres, one from the northern region and one from the eastern region)*	Stratified by healthcare model and operational districts (ODs) *(five ODs among five provinces: one referral hospital and three health centres from each OD)*
**Sample size**	66 GP practices	10 practices	5 referral hospitals and 15 health centers
**Weighted by**	# GPs per practice type in each regions	Not weighted	Not weighted
**Period**	2019-2020	2019-2020	2019-2020
**Representative**	For the 3 regions: Antwerp, Ghent, Kempenland	For central and northeast region of Slovenia.	For the healthcare intervention (NCDs clinic, WHO PEN and MoPoTso network)
