# Supplementary material for: Hypertension Cascade Across Three Healthcare Systems and in Relation to the Level of Implementation of the Integrated Care Package
Source: Int J Integr Care. 2025 Aug 22;25(3):22. doi: 10.5334/ijic.8921 (PMC12372687; doi:10.5334/ijic.8921)
Supplement: S4.a. — The ICP Grid scores of Belgium. [file ijic-25-3-8921-s6.pdf]

S4.a . The ICP Grid scores of Belgium

	1.Identification		2.Treatment		3.Education		4.Self-management		5.Collabaration		6.Organization		Overall	
type of practice	mean	sd	mean	sd	mean	sd	mean	sd	mean	sd	mean	sd	mean	sd
monodisciplinary - FFS	4.31	0.25	3.65	0.38	0.00	0.00	2.01	0.59	2.36	0.73	2.25	1.09	2.43	0.51
Multidisciplinary - FFS	4.32	0.24	3.64	0.31	0.86	0.87	2.21	0.51	2.59	0.67	2.69	1.01	2.72	0.60
Multidisciplinary - capitation	4.36	0.19	3.63	0.33	0.70	0.87	2.17	0.49	2.45	0.62	2.52	1.07	2.64	0.60
Weighted total[a]	4.27	0.77	3.57	0.64	0.14	0.05	1.60	0.25	1.96	0.33	1.56	0.28	2.18	0.39

Notes: *[a] the average of the stratum (=region) specific averages (this allows us to take the different distribution of practice types per region into account).*
