# Supplementary material for: Hypertension Cascade Across Three Healthcare Systems and in Relation to the Level of Implementation of the Integrated Care Package
Source: Int J Integr Care. 2025 Aug 22;25(3):22. doi: 10.5334/ijic.8921 (PMC12372687; doi:10.5334/ijic.8921)
Supplement: S4.b. — The ICP Grid scores of Slovenia. [file ijic-25-3-8921-s7.pdf]

S4.b . The ICP Grid scores of Slovenia

	1.Identification		2.Treatment		3.Education		4.Self-management		5.Collabaration		6.Organization		Overall	
Region	mean	sd	mean	sd	mean	sd	mean	sd	mean	sd	mean	sd	mean	sd
urban	5.00	0.00	3.97	1.09	4.22	0.72	2.26	1.86	3.00	1.89	3.69	1.65	3.69	1.20
rural	4.88	0.23	3.97	1.10	4.13	0.61	2.88	1.68	3.10	1.82	3.58	1.92	3.76	1.23
Total	4.94	0.12	3.97	1.10	4.17	0.61	2.57	1.77	3.05	1.85	3.64	1.78	3.72	1.21

Notes: *Scores for regions were calculated as means of scores for appropriate health centres.*
