# Supplementary material for: Hypertension Cascade Across Three Healthcare Systems and in Relation to the Level of Implementation of the Integrated Care Package
Source: Int J Integr Care. 2025 Aug 22;25(3):22. doi: 10.5334/ijic.8921 (PMC12372687; doi:10.5334/ijic.8921)
Supplement: S4.c. — The ICP Grid scores of Cambodia. [file ijic-25-3-8921-s8.pdf]

S4.c. The ICP Grid of Cambodia

		1.Identification		2.Treatment		3.Education		4.Self-management		5.Collabaration		6.Organization		Overall	
OD (Province)	Organisational type	mean	sd	mean	sd	mean	sd	mean	sd	mean	sd	mean	sd	mean	sd
OD Daunkeo (Takeo Province)	(a), (b), (c)	4.4	0.7	2.6	1.1	2	0.03	2.2	0.8	1.8	0.5	2.3	1.1	2.6	1.0
OD Kong Pisei (Kampong Speu Province)	(c)	4.3	0.8	2.1	1.0	1.1	0.6	2.2	0.8	2.4	0.6	2.8	0.9	2.5	1.0
OD Sort Nikum (Siem Reap Province)	(a), (b)	4.1	0.6	2.4	1.3	1.4	0.4	1.9	1.0	1.3	0.8	2.2	1.3	2.2	0.9
OD Pearaing (Prey Veng Province)	(a), (b)	4.1	0.8	2.4	1.0	1.6	0.3	2	1.0	1.4	1.0	2.0	1.4	2.3	0.9
OD Samrong (Oddormeanchey Province)	(a)	4.0	0.7	2.5	1.0	1.6	0.2	1.9	0.9	1.3	0.8	2.3	1.0	1.3	0.9
Total		4.2	0.2	2.4	0.7	1.5	0.3	2.0	0.1	1.6	0.4	2.8	0.3	2.2	0.5

Notes: *(a) Hospital-based care; (b) Health center-based care; (c) Community-based care*
