# Supplementary material for: Hypertension Cascade Across Three Healthcare Systems and in Relation to the Level of Implementation of the Integrated Care Package
Source: Int J Integr Care. 2025 Aug 22;25(3):22. doi: 10.5334/ijic.8921 (PMC12372687; doi:10.5334/ijic.8921)
Supplement: S5. — Information about the Focus Group Discussions and the respondents. [file ijic-25-3-8921-s9.pdf]

S5. Information about the Focus Group Discussions and the respondents

FGD	Date	Duration	Respondent	Gender	Expertise/discipline
Belgium	18/04/2023	1h.31	PB	M	Health sociology and data science, health data and health information system
			JVO	F	Public Health, health systems family medicine
			KD	F	Family medicine, primary care
			Extern: JVH	M	Epidemiologist, chronic diseases, HIS and BELHES, quality of care
Cambodia	07/042023	1h.39	SC	F	Pharmacy, epidemiology
			SC	M	Epidemiology, chronic diseases
			IP	M	Health systems and policy, medicine, chronic diseases
			Extern: KS	M	Health policy, medicine, public health
Slovenia	23/03/2023	1h.10	ČZ	M	Family medicine
			ZKK	F	Family medicine, integrated care, epidemiology
			Extern: LL	F	Family medicine, chronic care
