# Supplementary material for: Hypertension Cascade Across Three Healthcare Systems and in Relation to the Level of Implementation of the Integrated Care Package
Source: Int J Integr Care. 2025 Aug 22;25(3):22. doi: 10.5334/ijic.8921 (PMC12372687; doi:10.5334/ijic.8921)
Supplement: S6. — Country and gender specific HTN cascade. [file ijic-25-3-8921-s10.pdf]

S6. Country and gender specific HTN cascade

		In care		In treatment		Adhered to treatment		Under control	
		%	ASR	%	ASR	%	ASR	%	ASR
Men	Belgium	92.31	89.29	86.34	81.38	74.04	68.81	40.94	43.89
	Cambodia	82.04	77.03	80.00	75.26	42.45	40.36	8.16	9.13
	Slovenia	56.11	54.84	55.45	53.97	40.65	39.75	20.00	19.45
Women	Belgium	95.97	94.86	89.43	85.57	78.63	69.48	52.31	37.36
	Cambodia	87.58	83.64	86.97	82.99	50.42	44.70	35.83	31.63
	Slovenia	58.24	54.17	57.73	53.51	44.72	41.77	24.97	23.79

Note: *ASR= Age standardised Rate*
