# Supplementary material for: Hypertension Cascade Across Three Healthcare Systems and in Relation to the Level of Implementation of the Integrated Care Package
Source: Int J Integr Care. 2025 Aug 22;25(3):22. doi: 10.5334/ijic.8921 (PMC12372687; doi:10.5334/ijic.8921)
Supplement: S7. — Description of the determinants in the sample of the diagnosed HTN patients. [file ijic-25-3-8921-s11.pdf]

S7. Description of the determinants in the sample of the diagnosed HTN patients

	Belgium		Cambodia		Slovenia[c]	
	N[a]	%[b]	N	%	N	%
**Total N 'Diagnosed'**	1427		1074		22834	
**Gender**		100		100		100
men	696	48.4	245	25.3	11118	48.7
women	731	51.6	829	74.7	11716	51.3
**Age**		100		100		100
40-60	576	42.5	467	43.7	6298	27.6
60-69	456	30.1	369	31.5	9043	39.6
70-79	395	27.4	238	24.8	7493	32.8
**Education**		100		100		100
primary school or lower	183	11.3	954	88.2	636	19.1
lower secondary	293	21.9	96	9.2	861	25.8
higher secondary or higher	900	66.8	24	2.5	1839	55.1
**Financial situation**				100		100
poor	523	35.4	403	33.4	62	1.7
moderate	343	23.9	211	20.3	469	13.2
high	528	40.7	460	46.3	3027	85.1
**BMI**		100		100		100
< 25	345	25.2	684	63.6	2267	15.9
25-29.9 (overweight)	588	40.5	314	29.9	5757	40.4
>=30 (obese)	472	34.3	75	6.5	6231	43.7
**Smoking**		100		100		100
yes	208	17.3	221	18.5	2084	17.5
no	1004	82.7	853	81.5	9836	82.5
**T2D comorbidity**		100		100		100
no	1167	82.9	897	81.3	9884	75.7
yes: diabetes	260	17.1	177	18.7	3178	24.3

***Notes**: [a] sample frequencies; [b] proportions weighted to be representative at the level of the Belgian population; [c] for all the determinants (except for gender and age there is only information registered if the HTN patients are registered in HTN care*
